# Lifelong exercise is associated with more homogeneous motor unit potential features across deep and superficial areas of vastus lateralis

**DOI:** 10.1007/s11357-021-00356-8

**Published:** 2021-03-24

**Authors:** Eleanor J. Jones, Jessica Piasecki, Alex Ireland, Daniel W. Stashuk, Philip J. Atherton, Bethan E. Phillips, Jamie S. McPhee, Mathew Piasecki

**Affiliations:** 1 Clinical, Metabolic and Molecular Physiology, MRC-Versus Arthritis Centre for Musculoskeletal Ageing Research, National Institute for Health Research (NIHR) Nottingham Biomedical Research Centre, University of Nottingham, Nottingham, UK; 2 Musculoskeletal Physiology Research Group, Sport, Health and Performance Enhancement Research Centre, School of Science and Technology, Nottingham Trent University, Nottingham, UK; 3 Department of Life Sciences, Musculoskeletal Science and Sports Medicine Research Centre, Manchester Metropolitan University, Manchester, UK; 4 Department of Systems Design Engineering, University of Waterloo, Waterloo, Ontario Canada; 5 Department of Sport and Exercise Sciences, Musculoskeletal Science and Sports Medicine Research Centre, Manchester Metropolitan University, Manchester, UK

**Keywords:** Motor unit, Master athlete, Electromyography, Sarcopenia

## Abstract

Motor unit (MU) expansion enables rescue of denervated muscle fibres helping to ameliorate age-related muscle atrophy, with evidence to suggest master athletes are more successful at this remodelling. Electrophysiological data has suggested MUs located superficially are larger than those located deeper within young muscle. However, the effects of ageing and exercise on MU heterogeneity across deep and superficial aspects of vastus lateralis (VL) remain unclear. Intramuscular electromyography was used to record individual MU potentials (MUPs) and near fibre MUPs (NFMs) from deep and superficial regions of the VL during 25% maximum voluntary contractions, in 83 males (15 young (Y), 17 young athletes (YA), 22 old (O) and 29 master athletes (MA)). MUP size and complexity were assessed using area and number of turns, respectively. Multilevel mixed effects linear regression models were performed to investigate the effects of depth in each group. MUP area was greater in deep compared with superficial MUs in Y (*p*<0.001) and O (*p*=0.012) but not in YA (*p*=0.071) or MA (*p*=0.653). MUP amplitude and NF MUP area were greater, and MUPs were more complex in deep MUPs from Y, YA and O (all *p*<0.05) but did not differ across depth in MA (all *p*>0.07). These data suggest MU characteristics differ according to depth within the VL which may be influenced by both ageing and exercise. A more homogenous distribution of MUP size and complexity across muscle depths in older athletes may be a result of a greater degree of age-related MU adaptations.

## Introduction

Sarcopenia is the loss of muscle mass and function with age [3] and is the result of the combination of the atrophy and loss of individual muscle fibres [48]. It is associated with an increased risk of falls and a greater probability of fractures [50] and reduces the functional independence of older individuals and their ability to carry out activities of daily living [18]. As the proportion of older people in the population increases, with UK estimates of 12.2 million people over the age of 65 in 2018 [33], and with sarcopenia estimated to affect >50 million people over 60 globally [2], interventions to maintain musculoskeletal health in older adults at greater risk are more pressing than ever.

Lower limb muscles such as the quadriceps are important for mobility and balance [19] but often show the greatest reductions in muscle mass and strength with age and inactivity compared to other muscle groups [20, 24]; therefore, declines here have a greater detrimental functional impact. With age and inactivity, morphological changes within muscle fibres are observed including atrophy, reduced satellite cell number and reduced mitochondrial density [27, 31]. Alongside these changes, which contribute to the loss of strength, there are also neuromuscular changes observed at the single motor unit (MU) level. The term MU encompasses a motor neuron and the muscle fibres it supplies [6]. A reduction in MU number has been observed in multiple muscles [12, 40, 41] alongside increases in MU size with advancing age [22, 35, 39].

Data on the effects of lifelong exercise on the preservation of MUs are equivocal, with a study showing no difference between master athletes (MA) and recreationally active young in the tibialis anterior (TA) [42] but others showing no difference between MA and age-matched recreationally active controls in the TA [36], biceps brachii (BB) [41] and VL [37]. However, there are multiple lines of evidence on the process of fibre reinnervation suggesting it is more successful in highly active older adults possibly reducing overall fibre loss, demonstrated by intramuscular electromyography (iEMG) [37], fibre type grouping from biopsies [29, 52] and fewer histological markers of denervation [14, 44].

The process of axonal regeneration and synaptogenesis is achieved through collateral axonal sprouting and the formation of new neuromuscular junctions with denervated fibres, likely stimulated by neurotrophins which are increased following exercise [5, 16]. The increase of fibre type grouping in older human muscle, particularly of type 1 fibres [1, 15], has also been observed in animal models where it is attributed to a preference of a slow phenotype to reinnervate local fibres [9]. Whilst grouping indicates fibres are of the same type, it does not indicate they belong to the same MU and may not directly infer successful fibre reinnervation [28].

Previous research has demonstrated that MU size differs across muscle depth in young VL, with deeper MUs smaller than those located in superficial regions [17]. However, it is currently unknown if these findings are the same in older people and if this observed heterogeneity can be affected by activity levels. The aims of this study were to determine the heterogeneity of MUP features in deep and superficial regions of the vastus lateralis (VL) muscle and to determine the combined effects of ageing and lifelong exercise.

## Methods

### Participants

Eighty-three healthy male volunteers gave written informed consent to take part in the study approved by the Manchester Metropolitan University Research Ethics Committee and the National Research Ethics Service Committee Northwest (15/NW/0426). This included 32 young: 15 controls, 7 young endurance athletes, 10 young power athletes; and 51 old: 22 controls, 18 master endurance athletes, 11 master power athletes. These individuals were grouped into young (Y), young athletes (YA), old (O) and master athletes (MA), with characteristics summarised in Table 1. The controls were recreationally active and were recruited from the university population and local community. All of the athletic participants were competing in their respective sports at the time of testing, and all completed more than 5 h of training per week specific to their discipline. Athletes were recruited from running clubs, two national masters athletics competitions and from an advertisement in a national athletics magazine. All young athletes had trained specifically for their respective events for a minimum of 5 years prior to testing. All master athletes had trained specifically for their events since young adulthood (>18 years), and the median number of years of training at the point of testing was 46 years for the endurance athletes and 51 years for the power athletes. Direct comparisons of whole muscle function and MU potential size in these participants have previously been published [37].

**Table 1:** Mean (SD) participant characteristics for each group

	**Group**					
	**Y (*n*=15)**	**YA (*n*=17)**		**O (*n*=22)**	**MA (*n*=29)**	
Age (years)	26 (5)	27 (4)		70 (4)	70 (5)	
Height (m)	1.78 (0.07)	1.80 (0.04)		1.75 (0.06)	1.74 (0.06)	
Weight (kg)	72.3 (7.7)	79.7 (13.8)		73.9 (6.8)	68.9 (8.4)	
BMI (kg/m^2^)	22.9 (2.4)	24.6 (4.0)		24.3 (1.9)	22.7 (2.5)	
	**Y (*n*=15)**	**YE (*n*=7)**	**YP (*n*=10)**	**O (*n*=22)**	**ME (*n*=18)**	**MP (*n*=11)**
VL thickness (cm)	2.5 (0.4)	2.2 (0.2)	3.4 (0.5)	1.7 (0.4)	1.9 (0.5)	2.0 (0.4)
Body fat (%)	17.1 (6.3)	8.7 (3.6)	14.5 (4.9)	22.4 (5.4)	13.9 (5.5)	18.1 (5.9)

Young controls (Y), young athletes (YA), old controls (O), master athletes (MA), vastus lateralis (VL), young endurance athletes (YE), young power athletes (YP), master endurance athletes (ME), master power athletes (MP)

#### Anthropometric measures

Body mass and height were measured using calibrated scales and stadiometry, respectively, and body mass index (BMI) calculated. Information on the individual muscle cross-sectional area (CSA) and methods of recording VL thickness have been previously published [37]. In brief, VL thickness was measured in the right leg with magnetic resonance imaging (MRI) using a T1-weighted turbo 3D sequence on a 0.25-T G-Scan with the participants lying supine (Esaote, Genoa, Italy). Images were exported and analysed off-line as previously described, using Osirix imaging software (Osirix medical imaging, Osirix, Atlanta, GA, USA; [26]). Total body fat percentage was assessed by dual-energy X-ray absorptiometry (Lunar Prodigy Advance, version EnCore 10.50.086; GE Healthcare, UK) with legs and arms fully extended in the supine position.

### Experimental protocol

#### Strength assessment

Right knee extensor strength was assessed with participants sitting with hips and knees flexed at 90° and the leg securely fastened to a force transducer 30 cm below the centre of the knee joint. To familiarize with the equipment and to ‘warm-up’ the muscle, participants performed a series of submaximal contractions. They were then instructed to perform a maximal isometric contraction, accompanied by verbal encouragement and visual feedback of force on a computer screen. This was repeated three times, with 60-s rest intervals. The best effort was taken as a maximum voluntary isometric contraction force (MVC).

#### Surface EMG (sEMG)

An active recording sEMG electrode (disposable self-adhering Ag-AgCl electrodes; 95 mm^2^, Ambu Neuroline, Baltorpbakken, Ballerup, Denmark) was placed over the motor point located around the mid-thigh of the VL, identified using a cathode probe (Medserve, Daventry, UK) to apply percutaneous electrical stimulation at 400 V, pulse width of 50 μs and current of around 8 mA (DS7A Digitimer, Welwyn Garden City, Hertfordshire, UK). A self-adhesive electrode (Dermatrode, Farmadomo, NL) was used as the anode placed over the right gluteus. A reference electrode was placed over the patella tendon and a common ground electrode placed over the patella. The common ground electrode served for both surface and intramuscular EMG (iEMG) measurements. Surface EMG signals were bandpass filtered between 5 and 5 kHz via CED 1902 amplifiers (Cambridge Electronics Design Ltd., Cambridge, UK), sampled at 10 kHz and digitized with a CED Micro 1401 data acquisition unit (Cambridge Electronic Design).

#### Intramuscular EMG (iEMG)

A 25-mm concentric needle electrode (Model N53153; Teca, Hawthorne, NY) was inserted immediately adjacent to the recording surface electrode over the motor point, to a depth of approximately 10 to 25 mm into the VL, depending on muscle size. The initial depth of insertion was based on participants’ muscle size with deeper insertions for larger muscles and was withdrawn superficially between contractions. At each new location, the participant performed a low-level contraction to ensure MUPs were of sufficient sharpness (i.e. quality). During intramuscular needle insertions, it is possible to differentiate between subcutaneous fat tissue and muscle based on needle resistance during insertion and passing of the VL aponeurosis. This combined with inspection of the signal during low-level contractions ensures the needle tip remains within the muscle. All needle insertions and iEMG recordings were performed by the same investigator. The participant performed a voluntary, low force contraction whilst the needle position was adjusted to obtain intramuscular MUPs with peak second derivative values >5 kV/s^2^, thus ensuring the recording needle electrode was close to fibres belonging to the sampled MUs [34, 45]. The participant then performed a voluntary contraction lasting 12–15 s, aiming to hold a target line set at 25% MVC shown on a computer monitor, and rested for approximately 30 s between contractions. The needle electrode was then repositioned by combinations of rotating the bevel 180° and withdrawing it by 10–25 mm, dependent on muscle size. The procedure of needle positioning, voluntary contraction and signal recording was repeated until a minimum of six recordings from varying depths from deep to superficial had been obtained to sample from representative sets of MUs. To clearly differentiate the effects of depth and reduce the probability of repeat MUP recordings from different perspectives of the same MU, here, we report data from the deepest and most superficial recordings only. iEMG signals were bandpass filtered from 10 to 10 kHz and sampled at 25 kHz. The force and EMG signals were displayed in real-time using Spike2 software (v8.01), and data were stored for off-line analysis.

### Data analysis

Deep motor units were defined as those recorded at the first depth the needle electrode was positioned, for both orientations (0° and 180°). Superficial MUs were those recorded at the final depth the needle was positioned, following needle withdrawal, for both orientations. Again, a low force contraction was performed to examine signal quality following each change of depth and orientation.

#### EMG analysis

The procedures for recording and analysing individual MUPs and surface motor unit potentials (sMUPs) have been described in detail previously [36, 40]. Intramuscular and surface EMG signals were analysed using decomposition-based quantitative electromyography (DQEMG) [46]. DQEMG was used to automatically identify MUPs and their corresponding sMUPs. Individual MUPs from MU potential trains (MUPTs) of separate MUs were identified from the iEMG signal. MUPTs that were composed of MUPs from more than one MU or had fewer than 40 MUPs were excluded. Individual MUP times of occurrence were used as ‘triggers’ to estimate a corresponding sMUP, thereby providing surface representations of deep and superficial MUs sampled during the 25% MVC contractions. All MUP and sMUP templates were visually inspected and their markers adjusted, where required, to correspond to the onset, negative-phase onset (sMUP only), end and positive and negative peaks of the waveforms.

MUP amplitude was measured from the maximal positive and negative peaks, and the area was taken as the total area within the MUP duration (onset to end) and is indicative of MU size. The MUP complexity was assessed using the number of turns in the MUP template. A ‘turn’ was defined as a change in direction of the waveform of at least 25 μV. The number of turns in the MUP templates indicates the level of temporal dispersion across individual muscle fibre contributions to a single MUP. MUP amplitude was divided by sMUP amplitude to give a MUP:sMUP ratio, providing a direct comparison of MUPs measured at the skin surface and intramuscularly across depths.

A near fibre MUP (NFM) is defined as the acceleration of its corresponding MUP and calculated by applying a second-order, low-pass differentiator to the MUP which effectively reduces the recording area of the needle electrode to within ∼350 μm, thereby ensuring only the closest fibres significantly contribute to the NFM and reducing interference from distant active fibres of other MUs [34, 45].

#### Statistical analysis

Multilevel mixed effects linear regression models were performed in StataSE (v15.0, StataCorp LLC, TX, USA). As no interaction effects were observed between group and depth, separate models were performed for each group to account for within-subject variability and test for differences with depth in MUP features. Significance was assumed if *p*<0.05. Additionally, regression coefficients and 95% confidence intervals (CI) are reported.

## Results

### Participant characteristics

There were no depth-related differences in MUP and NFM features between the power and endurance athletes within each age group, so these were grouped accordingly. Participant characteristics are shown in Table 1. The mean age-graded performance (AGP) of the young athletes in their respective disciplines was 77.6 ± 2.3, and for master athletes, AGP was 81.2 ± 9.6, representing very high levels of athletic ability. A total of 1414 MUs were sampled, with a total mean of 17 ± 7 per person, comprising 10 ± 5 deep MUs and 7 ± 5 superficial MUs.

### Depth variation

#### Motor unit potential features

The ratio between the MUP and sMUP amplitude was significantly greater for deep MUs in all groups (Y: β=−1.06; CI=−1.52: −0.602; *p*<0.001; YA: β=−1.11; CI=−1.55: −0.67; *p*<0.001; O: β=−0.695; CI=−1.34: −0.051; *p*=0.034; MA: β=−0.865; CI=−1.45: −0.283; *p*=0.004; Table 2), meaning that for deep MUs, the size of an sMUP less closely matched its corresponding MUP than for superficial MUs.

**Table 2:** Regression coefficient (β) and 95% confidence intervals (CI) for all motor unit parameters

	Regression coefficient (β)	Confidence Interval (CI)	Significance (*p*)
MUP features			
MUP amplitude			
Y	−160	−234: −85.5	*p*<0.001
YA	−93.4	−179: −8.17	*p*=0.032
O	−179	−321: −35.9	*p*=0.014
MA	−61.2	−151: 28.6	*p*=0.182
MUP area			
Y	−316	−462: −170	*p*<0.001
YA	−154	−321: 13.4	*p*=0.071
O	−292	−521: −62.9	*p*=0.012
MA	−45.5	−244: 153	*p*=0.653
Turns			
Y	−0.588	−0.975: −0.201	*p*=0.003
YA	−0.430	−0.747: −0.114	*p*=0.008
O	−0.696	−1.19: −0.2	*p*=0.006
MA	−0.062	−0.397: 0.273	*p*=0.716
MUP:sMUP amplitude			
Y	−1.06	−1.52: −0.602	*p*<0.001
YA	−1.11	−1.55: −0.67	*p*<0.001
O	−0.695	−1.34: −0.051	*p*=0.034
MA	−0.865	−1.45: −0.283	*p*=0.004
NFM features			
NFM area			
Y	-1.03	−1.64: −0.42	*p*=0.001
YA	-0.88	−1.54: −0.22	*p*=0.009
O	-1.36	−2.35: −0.379	*p*=0.007
MA	-0.71	−1.50: 0.078	*p*=0.077

Multilevel mixed effects linear regression model outputs for young controls (Y), young athletes (YA), old controls (O), master athletes (MA), motor unit potential (MUP), surface motor unit potential (sMUP), near fibre motor unit potential (NFM)

**Fig. 1:**
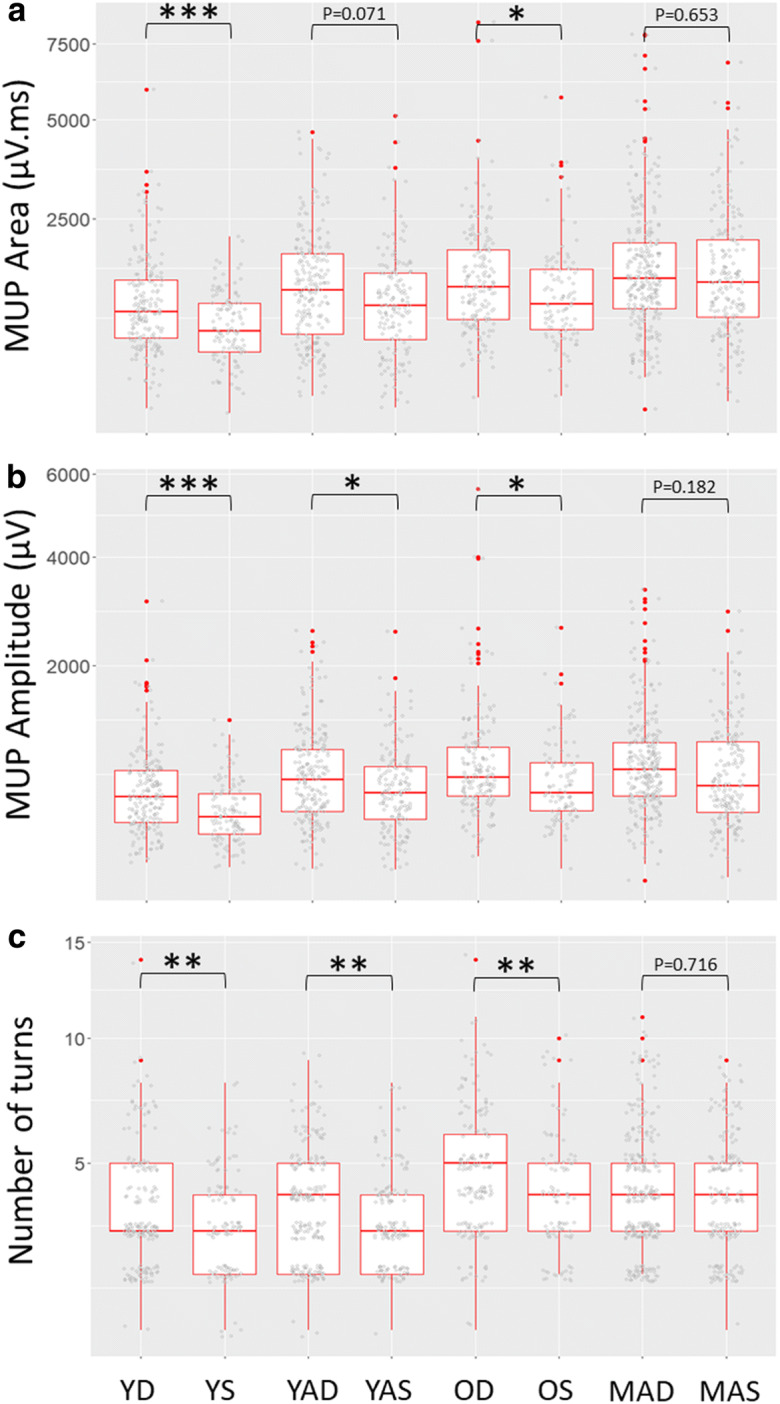
Individual data points and box plots (median + 25th/75th percentile) for motor unit potential (MUP) area (**a**), MUP amplitude (**b**) and discrete number of turns (**c**) for young controls (Y), young athletes (YA), old controls (O) and master athletes (MA) for deep (D) and superficial (S) MUs of the vastus lateralis. *p<0.05, **p<0.01. All MUs presented for data visualisation however statistical analyses are based on multilevel mixed effects linear models to account for data clustering

MU potential area was significantly greater for deep MUs in the Y (β=−316; CI=−462: −170; *p*<0.001) and O (β=−292; CI=−521: −62.9; *p*=0.012) but was not significantly different between deep and superficial MUPs for YA (β=−154; CI=−321: 13.4; *p*=0.071), and no difference for MA (β=−45.5; CI=−244: 153; *p*=0.182; Table 2; Fig. 1a). Similarly, MUP amplitude was significantly greater for deep MUs compared to superficial MUs in Y (β=−160; CI=−234: −85.5; *p*<0.001), YA (β=−93.4; CI=−179: −8.17; *p*=0.032) and O (β=−179; CI=−321: −35.9; *p*=0.014), but no differences were observed between depths in MA (β=−61.2; CI=−151: 28.6; *p*=0.182) (Table 2; Fig. 1b). MUP complexity, as assessed by the number of turns, was greater for deeper MUs compared to superficial in Y (β=−0.588; CI=−0.975: −0.201; *p*=0.003), YA (β=−0.43; CI=−0.747: −0.114; *p*=0.008) and O (β=−0.696; CI=−1.19: −0.2; *p*=0.006), but no differences were observed between depths in MA (β=−0.062; CI=−0.397: 0.273; *p*=0.716) (Table 2; Fig. 1c).

#### Near fibre motor unit features

**Fig. 2:**
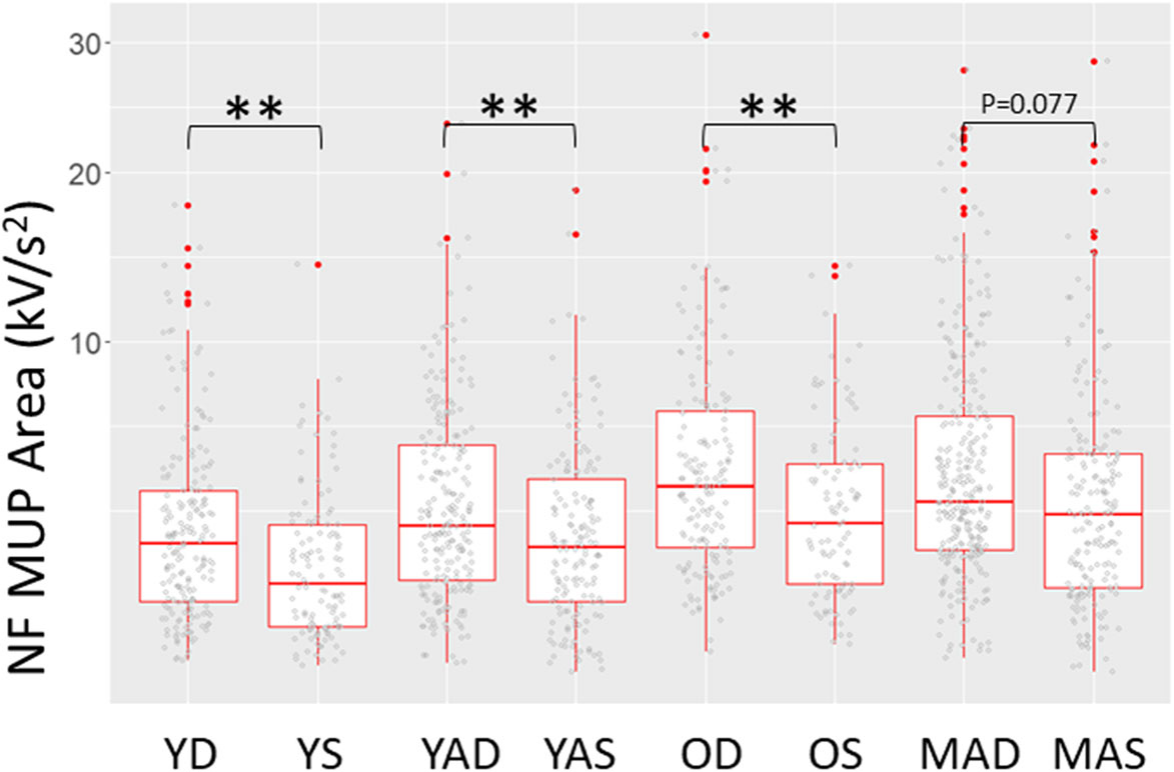
Individual near fibre motor unit potential (NF MUP) areas and box plots (median + 25th/75th percentile) for young controls (Y), young athletes (YA), old controls (O) and master athletes (MA) for deep (D) and superficial (S) MUs of the vastus lateralis. **p<0.01. All MUs presented for data visualisation however statistical analyses are based on multilevel mixed effects linear models to account for data clustering

NFM area was significantly greater for deep MUs in Y (β=−1.03; CI=−1.64: −0.42; *p*=0.001), YA (β=−0.88; CI=−1.54: −0.22; *p*=0.009) and O (β=−1.36; CI=−2.35: −0.38; *p*=0.007) but not MA (β=−0.71; CI=−1.50: 0.078; *p*=0.077) (Table 2; Fig. 2).

## Discussion

Here, we demonstrate that MUP features of the VL differ according to muscle depth, and these differences are further influenced by age and activity level. We found that key parameters indicative of MU size and complexity were greater in deep MUs than those located superficially in healthy young participants. A similar heterogeneous distribution of MUP features was also shown in the young athletes and old control groups. Conversely, master athletes demonstrated a more homogenous distribution of MUP features across deep and superficial recording sites with all MUP features showing no differences across depth.

Spike triggered averaging was used here to examine the surface representation of MUPs, from deep and superficial MUs. Our findings from the ratio of MUP amplitude to sMUP amplitude indicate that the contribution of deep MUs to the sMUP is significantly smaller than those of superficial MUs. This finding adds further evidence that surface EMG recording will preferentially record from superficial MUs, and as such may not be representative of the entire muscle pool [30]. Signals from sEMG alone are attenuated by subcutaneous fat and connective tissue [7, 32], and here, we demonstrate that this occurs in all population groups (via the MUP: sMUP amplitude ratio), inclusive of athletes who have much lower body fat levels than age-matched controls.

Our results show that MUs sampled from deeper regions of VL generated larger NFM area and MUP amplitude in Y, YA and O. One possibility is that deep VL MUs are structurally distinct from those in superficial regions, which are similar to observations of MUPs in the trapezius muscle [13]. However, the trapezius muscle is structurally different from the VL, and in the VL, individual fibres may span the entire deep to superficial aponeuroses [10]. Therefore, factors other than distinct MU pools may influence the differences in MUP features between deep and superficial regions, including localised intramuscular connective and/or fat tissue, or may be related to the heterogeneity of fibre CSA along their full length [25].

These findings differ from previous reports where deep MUs generated smaller MUPs [17] in healthy young VL. Key differences in recording technique could possibly explain these differences. At the same contraction intensity, MUP and NFM measures provide a more accurate view of MU activity than those recorded by surface methods more often used [17, 38]. This concept is also evidenced here with the weaker relationship between deep MUP and sMUP amplitude in all groups. Although the exact depth of the detection area of the recording electrode within the VL was not measured, we can confidently differentiate between deep and superficial regions as the needle was incrementally withdrawn between recordings. We made efforts to ensure the needle electrode did not pass the deeper VL aponeurosis, inserting very little of the 25-mm needle into the smallest muscles (VL thickness ranged between 8.2 and 36.6 mm in our cohort). Findings from Knight and Kamen [17] were applied to depths of 47 mm at an angle perpendicular to the skin, which would exceed the depth from even our young power athletes, and therefore may have been sampling from vastus intermedius.

The physiological importance of the differences we observed in MUP features across depths are unclear; nevertheless, they have methodological implications when recording MUs. In seminal work from Lexell et al. [21], autopsied sections of VL indicated a more even distribution of fibre size across depths of old muscle when compared to young, which could influence the size of the MUP. The size of an MUP cannot directly infer the size of the MU in terms of innervation ratio but is indicative of it [34, 51]. The MUP area and amplitude are also influenced by fibre CSA and proximity of the MU fibres to the recording electrode and estimating the relative contribution of each is difficult in vivo [38]. By applying a second-order differentiator filter to all sampled MUPs, we are able to assess the acceleration of fibre de/repolarisation and ensure the electrode is close to some fibres of an MU [45, 46], serving as an aspect of quality control.

Master athletes demonstrated a different relationship of MUP features across depth in comparison to the other groups. MUP area and amplitude showed no significant differences across the depth and neither did NFM area. These findings demonstrate a more homogenous distribution of MU fibres across the VL which may be a result of a decrease in MU size in deep MUs or an increase in size of superficial MUs, or a more homogenous distribution of non-contractile material and/or fibre CSA. As previously reported in this cohort, overall MUPs were larger in MA [37], and this provides further evidence that MU remodelling has occurred to a greater extent in the life-long exercisers. Previous studies have found increased MUP area in older people [12, 40], but in MAs, larger MUPs were found compared to age-matched controls [37]. The number of turns, representing MUP complexity, also showed no differences across depth in this MA group, further indicating MU structural and/or functional similarities across depth. Increases in MUP complexity have been shown to be a pathological feature in both myopathies and neurogenic disorders [8].

Plasticity of the neuromuscular system is important to enable regrowth and repair of motor neurons supplying muscle fibres, which ultimately prevents or minimises their loss. Previous histological findings have shown a limited capacity for reinnervation in non-athletic older people when compared to age-matched highly active counterparts [29, 44]. In combination with depth differences in other groups, the current findings of a more homogenous distribution of MU fibres at a mid-level contraction intensity in MA could also support data findings of greater type 1 fibre remodelling and type 1 fibre grouping observed with age [11, 15]. The exact mechanisms responsible for this process are still to be elucidated but axonal sprouting can be partly attributed to increased exercise-induced neurotrophins and hormones [47, 5]. This therefore demonstrates the beneficial effects of exercise particularly in an older population, in preserving musculoskeletal structure and function, thereby promoting healthy ageing.

### Limitations

Although we did not have equal numbers of power and endurance athletes, in the YA and MA groups, we observed no differences in depth effects on MUP features; therefore, they were grouped for further analysis. Previous data has shown that both master endurance and power athletes demonstrate greater remodelling than age-matched controls [37]. This implies that the level of exercise is more relevant than type, particularly when aiming to prevent fibre loss through reinnervation in older people. However, differences between power and endurance athletes are frequently observed in muscle fibre type composition, size and strength [23, 49], so there can be limitations to grouping different modalities, especially when comparing functional measures such as power. Despite only males participating in this study, recent findings showed similar age-related trajectories of TA MU remodelling in males and females, suggesting the sex-based response to age is similar [35]. We also only sampled MUs at a single contraction intensity which does not reveal where in the MU pool changes are more prominent. It is possible that at different contraction intensities, differences in MUP features between O and MA would be more prominent, but age-related differences in MU properties are not affected by contraction intensity per se [4]. Nonetheless, 25% MVC represents a mid-level contraction that likely recruits from a pool similar to that commonly used during everyday activities such as walking [43].

## Conclusions

In conclusion, there is a heterogeneous distribution of MUP features in VL with those in deeper regions being larger and more complex when recorded during a mid-range contraction intensity. Other than the MUP area in YA, these are consistent across young and older recreationally active people. However, endurance and power master athletes show a more homogenous distribution, with similar MUP features across depths in the VL muscle. These differences may relate to MU structural differences across depth within the older active muscle, or they may indicate that MUs in older active muscle have undergone greater levels of remodelling resulting in increased fibre reinnervation, thereby altering MUP features. Overall, these findings support the notion that maintaining activity levels in older age promotes MU remodelling and fibre reinnervation which could ultimately be protective against the loss of muscle mass observed in ageing.

## Data Availability

The data that support the findings of this study are available from the corresponding author upon reasonable request.
